# Practical Departments

**Published:** 1895-02-09

**Authors:** 


### PRACTICAL DEPARTMENTS.

### TEA INFUSER.

For the speedy obtaining of a single and excellent cup of tea, the useful little apparatus which goes by the name of the "Unicus," patented by Mr. H. J. Cooper, of Thavie's Inn, E.C., will be found the best possible medium. It consists of a perforated strainer or cover, which can be fitted on to any ordinary teaspoon, inside which the tea leaves are placed and the spoon held in the cup while it is filled up with boiling water, and allowed to remain just according to the strength of the infusion required. Thus a single cup can be made in a minute without the necessity for a teapot, and on the most approved hygienic system, for by the prompt removal of the infuser, there is no danger of getting "extract of tannin" instead of tea.

Nurses should find this little infuser a great comfort, enabling them as it does to make for themselves that "cheering cup" to which they are so proverbially attached in the most convenient and satisfactory way. For busy people such little devices are of infinite service, for every simplification is a help, and a small comfort like this may oftentimes prove a very big boon when time and trouble are saved thereby at the right moment.

### ANOTHER HYGIENIC TEAPOT.

Mr. T. E. Williams, 21, King's Road, Brownswood Park, N., has come to the rescue of tea drinkers with yet another plan for providing them with an infusion so prepared as to render it perfectly free from all deleterious essences. It is contended that the teapot with a small infuser or container does not make the best tea, because the tea-leaves, being cramped together in a limited space, do not give out their best qualities, and so although the unpleasant and harmful effects which arise from the ordinary method of allowing the leaves to stew at the bottom of the pot are avoided, yet still the tea is not as good as it might be.

To obviate this objection the teapot invented by Mr. Williams is fitted with a perforated movable tray or strainer, the entire size of the pot, worked by a rod which passes through the centre of the pot and is manipulated from the top. To make the tea, the tray is allowed to rest upon the bottom of the teapot, the leaves are thrown in, and the boiling water poured over them in the usual way. In three or four minutes, when the desired strength has been attained, the strainer is pulled up above the level of the liquid by the centre rod, and held in position by a small lever. Thus it is claimed that the leaves have ample space to expand and give out all their goodness, while the infusion obtained is as free from tannic acid as that made in the infuser teapots which have become so well known of late. For cleaning purposes the whole top of the teapot can be taken off.

The only objection we can see is the slight clumsiness inseparable from any complication of apparatus. The system has everything to recommend it, and these teapots should find favour in the eyes of those who have much to say on the evil effects consequent on drinking tea which has "brewed," and become something little short of poisonous. The pots are made in several sizes, and can be obtained from Messrs. Sumerling and Co., 63, Bunhill Row, E.C., and Messrs. O'Brien, Thomas, and Co., 123, Queen Victoria Street, E.C.

